# Pantao Pill Improves the Learning and Memory Abilities of APP/PS1 Mice by Multiple Mechanisms

**DOI:** 10.3389/fphar.2022.729605

**Published:** 2022-02-25

**Authors:** Qiqi Xin, Weili Shi, Yan Wang, Rong Yuan, Yu Miao, Keji Chen, Weihong Cong

**Affiliations:** ^1^ Laboratory of Cardiovascular Diseases, Xiyuan Hospital of China Academy of Chinese Medical Sciences, Beijing, China; ^2^ National Clinical Research Center for Chinese Medicine Cardiology, Xiyuan Hospital, China Academy of Chinese Medical Sciences, Beijing, China

**Keywords:** cognitive impairment, oxidative stress, autophagy, apoptosis, neurotransmitter, pantao pill

## Abstract

**Background:** To explore the effect and mechanisms of Pantao Pill (PTP) on cognitive impairment.

**Methods:** Network pharmacology was performed to analyze the mechanism of PTP treating cognitive impairment. The targets of PTP and cognitive impairment were predicted and used to construct protein-protein interaction (PPI) networks. The intersection network was selected, and the core network was obtained through topological analysis. Enrichment analysis was conducted to obtain the GOBP terms and KEGG pathways. We then performed experiments to validate the results of the network pharmacology by using an APP/PS1 transgenic mouse model. The APP/PS1 mice were divided into four groups: the model group, the high-dose PTP (3.6 g/kg·d) group, the low-dose PTP (1.8 g/kg·d) group, and the positive control group (donepezil hydrochloride, 2 mg/kg·d). Wild-type (WT) C57 mice served as a normal control group. PTP and donepezil were administered by gavage for 8 weeks.

**Results:** Network pharmacology showed that PTP might improve cognitive impairment by regulating autophagy, apoptosis, and oxidative stress. For the Morris water maze test, a significant difference was shown in the total swimming distance among groups (*p* < 0.05) in the positioning navigation experiment, and with training time extension, the swimming speed increased (*p* < 0.01). In the space probe test, PTP administration significantly reduced the swimming path length and the escape latency of APP/PS1 mice (*p* < 0.05 or *p* < 0.01), whereas it had no effect on the swimming speed (*p* > 0.05). PTP (3.6 g/kg/d) rescued the reduction of norepinephrine and acetylcholine levels (*p* < 0.05), and increased the acetylcholinesterase concentration (*p* < 0.05) in the brain tissue. PTP (1.8 g/kg/d) increased the norepinephrine level (*p* < 0.01). PTP rescued the activity reduction of superoxide dismutase in the brain tissue (*p* < 0.01) and the neuron cell pyknosis in the hippocampal CA region (*p* < 0.05). PTP reduced ATG12 and PS1 expression (*p* < 0.05 or *p* < 0.01), and increased Bcl-2 expression in the brain tissue (*p* < 0.05).

**Conclusion:** PTP can significantly improve the learning and memory abilities of APP/PS1 mice, and the mechanism may be related to the increase of neurotransmitter acetylcholine and norepinephrine levels, the reduction of the excessive autophagic activation, and the suppression of oxidative stress and excessive apoptotic activity.

## Introduction

Cognitive impairment is one of the characteristics of human aging and often manifests as declines in attention, reasoning ability, learning ability, short-term and long-term memory, executive ability, and the perception of the surrounding environment (Kennedy et al., 2017). In China, the incidence of mild cognitive impairment alone is estimated to be as high as 15% among people over 60 years of age (Xue et al., 2018). Alzheimer’s disease (AD) is the most common cause of cognitive dysfunction, which has been reported to cause 50–75% of dementia cases. The number of AD patients in China is approaching 10 million. According to the Alzheimer’s Association Report 2021, more than 12 million people in the United States will suffer from Alzheimer’s and other dementias by 2050 (Alzheimer’s and association, 2021). The main pathological manifestations of AD are the formation of senile plaques caused by the deposition of amyloid β protein (Aβ) and the neurofibrillary tangling caused by the hyperphosphorylation of tau protein. The CA region of the hippocampus is a commonly affected area (Morrone et al., 2020). The occurrence of AD is related to a variety of pathological mechanisms. Mutations in the amyloid precursor protein (APP) and presenilin1 (PS1) genes can increase the production and accumulation of Aβ. The decrease in cellular autophagy leads to the accumulation of misfolded proteins and autophagosomes and further accelerate neurodegeneration (Nobili et al., 2021). Excessive Aβ deposition inhibits the activity of antioxidant enzymes, continuously enhances intracellular oxidative stress, and ultimately induces neuronal apoptosis (Chen et al., 2020).

Pantao Pill (PTP), is composed of traditional Chinese medicines including *Panax ginseng* C. A. Mey. [Araliaceae; Ginseng Radix et Rhizoma], *Asparagus cochinchinensis* (Lour.) Merr. [Liliaceae; Asparagi Radix], *Ophiopogon japonicus* (L. f). Ker-Gawl. [Liliaceae; Ophiopogonis Radix], *Lycium barbarum* L. [Solanaceae; Lycii Fructus], *Rehmannia glutinosa* Libosch. [Scrophulariaceae; Rehmanniae Radix], *Angelica sinensis* (Oliv.) Diels [Umbelliferae; Angelicae Sinensis Radix], *Alpinia oxyphylla* Miq. [Zingiberaceae; Alpiniae Oxyphyllae Fructus], *Ziziphus jujuba* Mill. var. *spinosa* (Bunge) Hu ex H.F. Chou [Rhamnaceae; Ziziphi Spinosae Semen], *Bombyx mori* Linnaeus [Bombycidae; Bombyx Mori Faeces], and *Juglans regia* L. [Juglandaceae; Juglandis Fructus Diaphragma]. PTP is an anti-aging prescription used by Chinese Emperor Qianlong of the Qing Dynasty and was previously awarded to officials who performed great feats, which now has been developed as Qinggong shoutao Pill. It can be used to treat dizziness, fatigue, memory decline, tinnitus, deafness, nocturia, and other diseases caused by aging. Clinical studies have shown that PTP induces significant improvements in amnestic mild cognitive impairment and can prevent 8.85% of amnestic mild CI patients from progressing to AD (Tian et al., 2019). PTP can significantly alleviate fatigue, dizziness, tinnitus, deafness, nocturia, and other clinical symptoms, reduce plasma lipid peroxide level, increase plasma estradiol and testosterone concentrations, and significantly improve transient and long-term memory in patients (≥45 years old) with manifestations of aging (Chen et al., 1984; Chen et al., 1985). PTP also inhibited the formation of lipid peroxide in rat liver homogenate *in vitro* and significantly increased the survival rate of quails (Chen et al., 1984; Chen et al., 1985). However, the mechanism by which PTP improves cognitive impairment in elderly patients is not fully understood. To further clarify the role and mechanism of PTP in improving cognitive impairment, this study used network pharmacology to predict its mechanism of action and further used an APP/PS1 double transgenic mouse model to explore the effect and mechanism of PTP on learning and memory abilities.

## Materials and Methods

### Network Pharmacology

The ingredients of PTP were retrieved from the Traditional Chinese Medicine Systems Pharmacology Database and Analysis Platform (TCMSP, https://tcmspw.com/tcmsp.php) and Traditional Chinese Medicines Integrated Database (TCMID, http://119.3.41.228:8000/tcmid/search/) databases (Xue et al., 2013; Ru et al., 2014). The absorption, distribution, metabolism, and excretion (ADME) system of the TCMSP database was used to select the active ingredients. The oral bioavailability ≥30%, drug-likeness ≥ 0.18, and blood brain barrier ≥ −0.3 were chosen as the boundaries, and the targets of the active ingredients were further predicted by the TCMSP and the Encyclopedia of Traditional Chinese Medicine (ETCM, http://www.tcmip.cn/ETCM/) databases (Xu et al., 2018). For components not included in the TCMSP database, the Bioinformatics Analysis Tool for Molecular mechanism of TCM (BATMAN-TCM) database was used to predict their active targets (Liu et al., 2016).

Effective targets of cognitive impairment were retrieved from the Online Mendelian Inheritance in Man (OMIM, https://www.omim.org/), the Pharmacogenomics knowledgebase (PharmGKB, https://www.pharmgkb.org/), the Genetic Association Database (GAD, https://geneticassociationdb.nih.gov/), and the Therapeutic Target Database (TTD, https://db.idrblab.org/ttd/) databases. Cytoscape 3.8 software was used to construct the PTP-drug-ingredient-target network and cognitive impairment-targets network. By using the Bisogenet plugin, the protein-protein interaction (PPI) network of PTP and related targets associated with cognitive impairment were further constructed, and the intersection of the two PPI networks was obtained. The intersection network was topologically analyzed with the CytoNCA plugin (Tang et al., 2015), and a new network was constructed by screening targets with a more than 2-fold increase in the median degree. Topological analysis was performed on the new network. The core network was built based on six topological features, including “degree centrality (DC)”, “betweenness centrality (BC)”, “closeness centrality (CC)”, “eigenvector centrality (EC)”, “network centrality (NC)”, and “local average connectivity-based method (LAC)”. All six algorithms are calculated for centralities and used to identify candidate targets. BC was calculated based on the total number of shortest paths from a target node to another node and the number of those paths that pass through a third node. CC determined a protein’s essentiality based on the number of the target node’s neighbors and the distance of the shortest path from the target node to another node. DC value was calculated based on the number of the target node’s neighbors and the weight of the edge connecting the node and another node. EC was calculated based on the eigenvector corresponding to the largest eigenvalue of the adjacency matrix. LAC determined a protein’s essentiality by evaluating the relationship between a protein and its neighbors, which was calculated based on the node-set containing all the neighbors of a target node. NC considered both the centrality of a node and the relationship between it and its neighbors, and a node’s essentiality was determined by the sum of the edge clustering coefficients of interactions connecting it and its neighbors.

The targets of the core network were enriched and analyzed by the Database for Annotation, Visualization and Integrated Discovery (DAVID, https://david.ncifcrf.gov/home.jsp) database to obtain Gene Ontology Biological Process (GOBP) and Kyoto Encyclopedia of Genes and Genomes (KEGG) information. In GO and KEGG prediction, the rich factor refers to the ratio of the number of target genes in the pathway to the number of all genes annotated. The larger the rich factor is, the greater the degree of enrichment. The *p*-value represents the significance of the focus gene enrichment. The GO and KEGG terms were considered for inclusion in outcomes as long as the corresponding *p* value was less than 0.05 and correlated to cognitive impairment pathology. The enrichment analysis results were visualized by the OmicShare platform (https://www.omicshare.com/tools/Home/Soft/getsoft) (Huang da et al., 2009).

### Drugs and Reagents

PTP refined powder (batch number P004) was provided by Darentang Pharmaceutical Factory (Tianjin, China). The preparation process of PTP refined powder is as below: the raw materials of the TCMs are mixed, dried, and crushed into fine powders; then, the fine powders are sieved and sterilized to be refined powder. The UPLC-LTQ-Orbitrap fingerprint results of PTP are shown in the Supplementary material (Supplementary Material S1). Donepezil hydrochloride (Arricent, national drug approval number H20050978) was purchased from Eisai Pharmaceutical Co., Ltd. (Shanghai, China). Malondialdehyde (MDA), superoxide dismutase (SOD), norepinephrine (NE), 5-hydroxytryptamine (5-HT), acetylcholine (Ach), and acetylcholinesterase (AchE) detection kits were purchased from Nanjing Jiancheng Institute of Biological Engineering (Nanjing, China). B-cell lymphoma 2 (Bcl-2) antibodies were purchased from Abcam (Shanghai) Trading Co., Ltd. (Shanghai, China). Antibodies against PS1, autophagy-related protein-12 (ATG12), beclin-1, glyceraldehyde-3-phosphate dehydrogenase (GAPDH) were purchased from CST (Shanghai) Biological Reagents Co., Ltd. (Shanghai, China). Horseradish peroxidase-labeled goat anti-mouse and goat anti-rabbit antibodies were purchased from Shanghai Beyotime Biotechnology Co., Ltd. (Shanghai, China).

### Instruments

The Morris water maze video tracking analysis system 2.0 was purchased from Chengdu Taimeng Technology Co., Ltd. (Chengdu, China). The automatic microplate reader was purchased from BioTek Instruments, Inc. (Vermont, United States). The BH-2 optical microscope was obtained from Olympus Corporation (Tokyo, Japan). The HPIAS-1000 pathology image analyzer was obtained from Zhongke Company (Beijing, China).

### Animals and Groups

Mice were purchased from the Institute of Experimental Animals of the Chinese Academy of Medical Sciences. Mice were assigned to five groups, including the normal control group, the model group, the low-dose PTP group, the high-dose PTP group, and the positive control group. Nine-month-old APP/PS1 transgenic mice were used in the model group, and C57BL/6 wild-type (WT) mice were used in the control group. The low-dose PTP (1.8 g/kg/d), high-dose PTP (3.6 g/kg/d) and donepezil (2 mg/kg/d) groups were administered the indicated treatments by intragastric gavage for 8 weeks. The 1.8 g/kg/d, and 3.6 g/kg/d dose of PTP are equivalent to the amount of drug administered using the approved PTP 14 g/kg/d, and 28 g/kg/d dose in a 70-kg patient, respectively. The normal control group and the model group were administered an equal amount of normal saline.

### Morris Water Maze

The Morris water maze test was designed to evaluate the spatial learning and memory abilities of mice according to previous studies and included a 4-day positioning navigation experiment and a 1-day space probe test (Vorhees and Williams, 2006). The Morris water maze test was conducted in a round white pool 120 cm in diameter and 40 cm deep. During the training and the formal experiment, the references outside the maze remained unchanged. In the first 4 days of the navigation experiments, a platform with a diameter of 10 cm was placed in the target quadrant and hidden 1 cm underwater. The mice were placed in the water facing the wall of the pool. The time mice spent on finding the platform was recorded as the escape latency. If the platform was not found within 120 s, the mice were led to the platform and made to stand on the platform for 30 s to generate memory. On day 5, the space probe test was performed. Mice were placed in water, the automatic video recording system recorded the time (swimming duration) and swimming path length each mouse took to find the platform, and the spatial memory abilities of the mice were evaluated.

### Measurement of Cholinergic and Monoaminergic Neurotransmitters and Related Enzymes in Brain Tissue

The brains were quickly removed on an ice platform after the mice were sacrificed. The hippocampus and cerebral cortex tissues were quickly frozen with liquid nitrogen and stored at −80°C. The concentrations of Ach, AchE, NE, and 5-HT were determined according to the instructions of the kits.

The Ach content was determined by a colorimetric method. The brain tissue was homogenized with the extraction reagent at a ratio of 1:9 and centrifuged at 2500 rpm for 10 min, and the supernatant was retained. The substrate solution was added to the supernatant and mixed. After being incubated at room temperature for 15 min, the terminating solution was added to stop the reaction, and the chromogenic solution was added and mixed. After being incubated for 10 min, the absorbance value of each tube was measured at a wavelength of 550 nm.

The activity of AchE was determined by an optical method. The brain tissue was homogenized with normal saline at a ratio of 1:9 and centrifuged at 2500 rpm for 10 min, and the supernatant was retained. The substrate buffer solution and color application solution were added successively, mixed, and reacted at 37°C for 6 min. The inhibitor and transparent agent were added, mixed, and incubated for 15 min, and the absorbance value of each tube was measured at a wavelength of 412 nm.

The concentrations of NE and 5-HT were measured by enzyme-linked immunoassay. Brain tissue samples were added to enzyme-labeled wells that were precoated with NE or 5-HT monoclonal antibodies. After incubation, biotin-labeled NE or 5-HT antibodies was added, bound with streptomycin-HRP to form an immune complex. After incubation and washing, the unbound enzyme was removed, and the substrate was added to produce a blue color, which was converted to yellow under the action of an acid. The intensity of the color positively correlated with the concentration of NE or 5-HT in the sample.

### Measurement of the Oxidative Stress Index in Brain Tissue

A 10% brain homogenate was prepared to measure the MDA level and total SOD activity. The MDA level was detected by the thiobarbituric acid method. Tissue homogenate supernatant was transferred to the measuring tube, and equal amounts of standard material, anhydrous ethanol and tissue supernatant were added to the standard tube, blank tube and control tube, respectively. Appropriate amounts of detection reagent were added to all tubes successively and mixed well. The tubes were heated in a water bath at 95°C for 40 min, then cooled to room temperature, and centrifuged at 3000 rpm for 10 min. The absorbance of the supernatant was measured at 532 nm to calculate the concentration of MDA. The activity of SOD was determined by the hydroxylamine method. The supernatant of the tissue homogenate and distilled water were added to the measuring tube and the control tube, respectively, and the detection reagents were added successively. After the solution was mixed following incubated in a water bath at 37°C for 40 min, the absorbance was measured at a wavelength of 550 nm, and the total SOD activity was calculated.

### HE Staining

Brain tissues were fixed in 10% formaldehyde, dehydrated, paraffin-embedded, and sliced continuously at a thickness of 5 μm. After gradient dewaxing with xylene and ethanol, the paraffin sections were stained with hematoxylin for 5 min, washed with distilled water, differentiated with hydrochloric acid and ethanol for 30 s, soaked in a warm water bath for 5 min, and redyed with eosin for 5 min. After conventional dehydration, the slices were sealed with transparent neutral resin. The sections were randomly selected, observed, and photographed.

### Western Blotting

After the mice were sacrificed, the cerebral cortex was quickly removed on an ice platform. Precooled RIPA protein extraction reagent and a protease inhibitor were added to extract the proteins. The slurry was homogenized with an electric homogenizer and incubated on ice for 20 min, followed by ultrasonic oscillation and centrifugation. After measuring the protein concentration by the BCA method, RIPA buffer was used to adjust all samples to the lowest sample concentration, and the samples were denatured in a 95°C water bath for 5 min. An SDS-PAGE gel was prepared. After gelation was complete, sample loading, electrophoresis, and membrane transfer processes were performed. The membrane was blocked with 5% bovine serum protein and incubated with primary and secondary antibodies. An ECL luminescence kit was used for staining, photos were taken, and gray values were calculated.

### Statistical Analysis

The results are expressed as the mean ± SD. The variance among multiple groups was assessed by a one- or two-way analysis of variance with/without repeated measures followed by a post hoc test. SPSS 17.0 statistical software was used for data analysis. *p* < 0.05 was considered statistically significant.

## Results

### Network Pharmacological Analysis of the Mechanism by Which PTP Improves Cognitive Impairment

**FIGURE 1:**
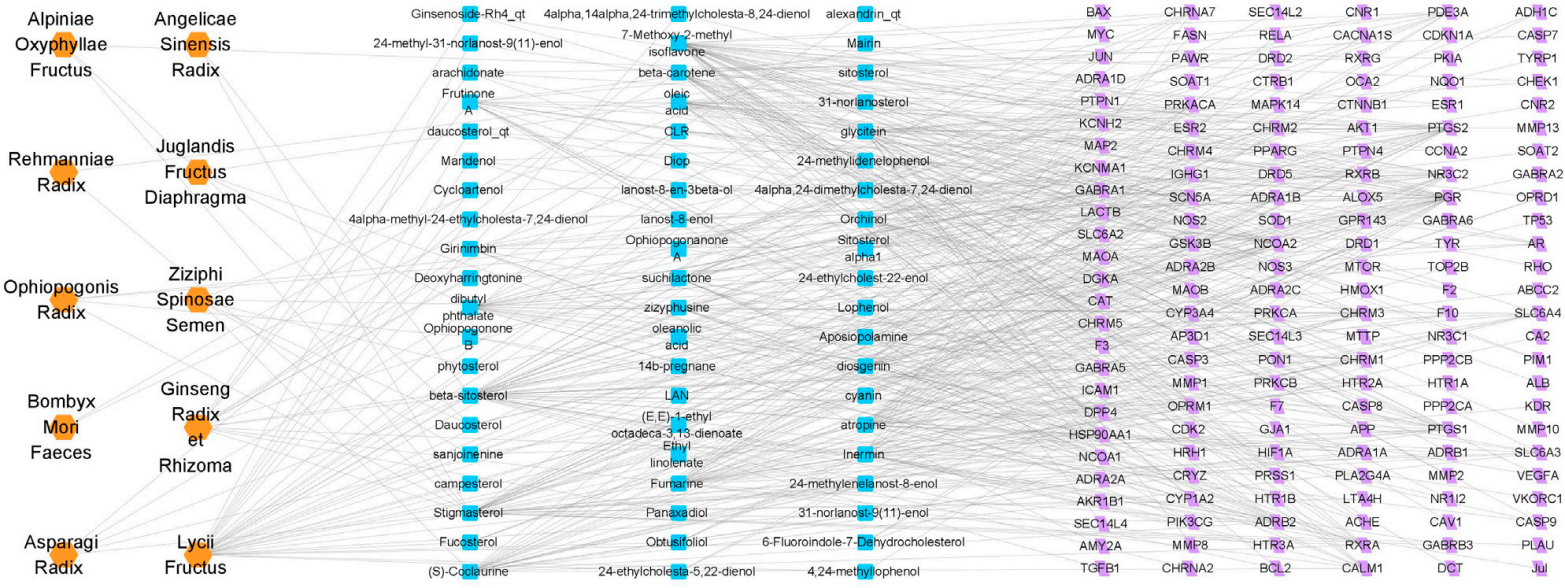
Drug-ingredient-target network of PTP. The orange nodes represent drugs, the blue nodes represent active ingredients, and the purple nodes represent possible targets of the active ingredients.

Sixty possible active components of PTP were identified (Table 1), and a total of 151 targets were obtained through prediction analysis (Figure 1). A total of 956 possible targets associated with cognitive impairment were retrieved (Supplementary Figure S2, Supplementary Material S3). The PTP target PPI network and the cognitive impairment target PPI network were constructed with the Bisogenet plugin (Supplementary Figure S3A, Supplementary Material S4). Then, the intersection of the PTP and cognitive impairment PPI networks was obtained (Supplementary Figure S3B, Supplementary Material S5). Topology analysis of the intersection PPI network was carried out, and a new network was constructed after screening with a DC > 48 as the threshold. Topology analysis of the new network was performed to obtain the core PPI network. Bounded by BC > 0.00021355, CC > 0.48908, DC > 124, EC > 0.013344, LAC >13.08955, NC > 14.446487, the core PPI network was built, which contained a total of 275 targets (Supplementary Figure S3C, Supplementary Material S5).

**TABLE 1:** PTP active ingredient prediction.

Drug	Ingredient	Drug	Ingredient
Alpiniae Oxyphyllae Fructus	sitosterol	Lycii Fructus	4,24-methyllophenol
	Stigmasterol		Lophenol
	Daucosterol		4alpha,14alpha,24-trimethylcholesta-8,24-dienol
Angelicae Sinensis Radix	beta-sitosterol		4alpha,24-dimethylcholesta-7,24-dienol
	Stigmasterol		4alpha-methyl-24-ethylcholesta-7,24-dienol
Asparagi Radix	beta-sitosterol		6-Fluoroindole-7-Dehydrocholesterol
	sitosterol		(E,E)-1-ethyl octadeca-3,13-dienoate
	Stigmasterol		lanost-8-en-3beta-ol
	diosgenin		lanost-8-enol
	7-Methoxy-2-methyl isoflavone		Obtusifoliol
Bombyx Mori Faeces	CLR	Ginseng Radix et	beta-sitosterol
	beta-carotene	Rhizoma	Stigmasterol
Juglandis Fructus	oleanolic acid		Fumarine
Diaphragma	dibutyl phthalate		Diop
	oleic acid		Inermin
Lycii Fructus	beta-sitosterol		Aposiopolamine
	Stigmasterol		Deoxyharringtonine
	CLR		arachidonate
	Sitosterol alpha1		Frutinone A
	Mandenol		Ginsenoside-Rh4_qt
	Ethyl linolenate		Girinimbin
	LAN		Panaxadiol
	Cycloartenol		suchilactone
	atropine		alexandrin_qt
	campesterol	Ophiopogonis	Stigmasterol
	cyanin	Radix	Ophiopogonanone A
	24-methylidenelophenol		Orchinol
	daucosterol_qt		Ophiopogonone B
	glycitein	Rehmanniae Radix	sitosterol
	14b-pregnane		Stigmasterol
	24-ethylcholest-22-enol	Ziziphi Spinosae	Mairin
	24-ethylcholesta-5,22-dienol	Semen	(S)-Coclaurine
	24-methyl-31-norlanost-9 (11)-enol		Daucosterol
	24-methylenelanost-8-enol		phytosterol
	Fucosterol		sanjoinenine
	31-norlanost-9 (11)-enol		zizyphusine
	31-norlanosterol		

**FIGURE 2:**
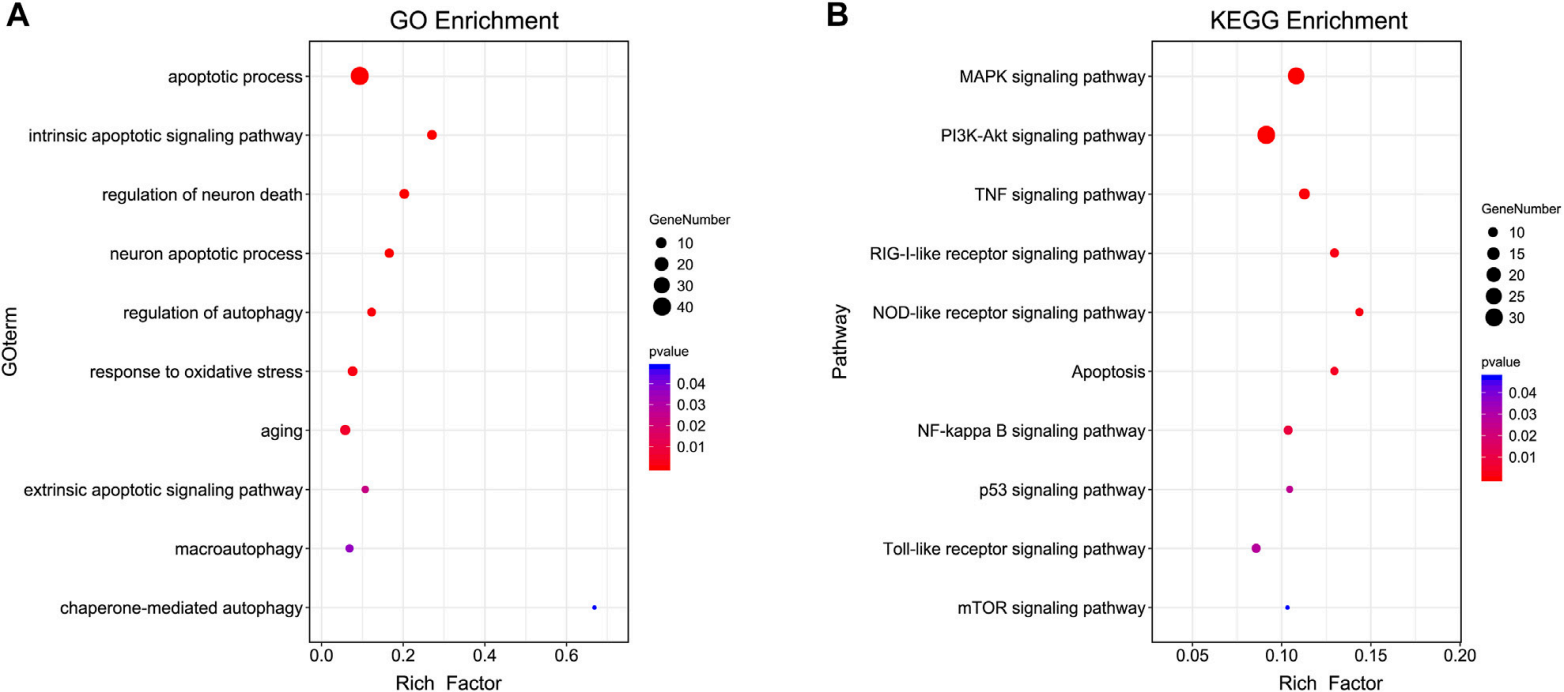
GOBP and KEGG analysis of core targets. **(A)** GOBP terms. **(B)** KEGG terms. The genes used for GO analysis were the core targets obtained from the top analysis of the merged PPI network. The *y*-axis represents GOBP or KEGG terms, the *x*-axis represents the rich factor, the dot size represents the number of genes, and the dot colors represent the *p*-value. The *p*-value represents the significance of the focus gene enrichment. The rich factor refers to the ratio of the number of target genes in the pathway to the number of all genes annotated. The larger the rich factor is, the greater the degree of enrichment.

In GO and KEGG prediction, both *p*-value and the rich-factor are essential indicators for possible mechanisms which could reflect the significance and degree of the target genes enriched in specific GO or KEGG terms. The GOBP enrichment analysis of the core PPI network targets indicated that PTP might play a role in improving cognitive impairment by acting on biological processes such as oxidative stress, apoptotic process, autophagy, aging, and neuron death (Figure 2A). The KEGG enrichment analysis showed that PTP might exert its cognitive improvement effects by regulating apoptosis, mTOR, PI3K-Akt, and MAPK signaling pathways (Figure 2B). In the present study, we verified some of the terms related to the pathology of cognitive impairment as examples with animal experiments.

### Effects of PTP on the Spatial Learning and Memory Abilities of APP/PS1 Transgenic Mice

**FIGURE 3:**
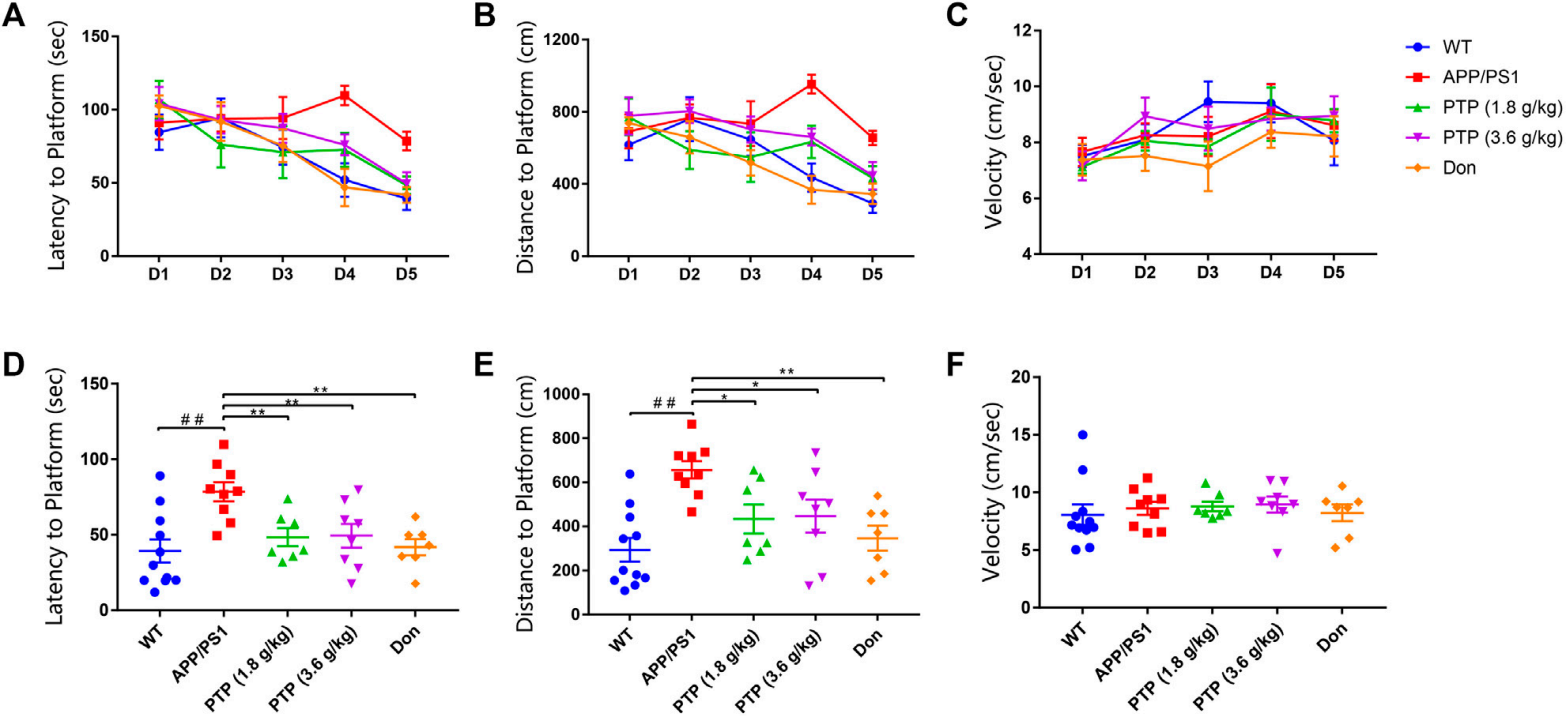
Evaluation of mice learning and memory in the Morris water maze. **(A)** Escape latency in the positioning navigation experiment. **(B)** Total swimming distance in the positioning navigation experiment. **(C)** Swimming speed to the platform in the positioning navigation experiment. **(D)** Escape latency to the platform in the space probe test. **(E)** Total swimming distance in the space probe test. **(F)** Swimming speed in the space probe test. Compared with the wild-type group: ^##^ *p* < 0.01; compared with the APP/PS1 group: ^*^ *p* < 0.05, ^**^ *p* < 0.01. (*n* ≥ 7).

There was a significant difference in the total swimming distance among groups (F = 3.287, *p* = 0.028) in the positioning navigation experiment. However, no difference was shown in swimming speed (F = 1.182, *p* = 0.344) or escape latency (F = 0.976, *p* = 0.439). With training time extension, the swimming speed showed an upward trend (F = 5.285, *p* = 0.009). The total swimming distance (F = 1.014, *p* = 0.409) and escape latency (F = 2.890, *p* = 0.064) showed downward trend, however, these trends were not statistically significant (Figures 3A–C).

For the space probe test, the escape latency (*p* < 0.01) and total swimming distance (*p* < 0.01) of the APP/PS1 group were significantly longer than those of the WT group. Compared with the model group, PTP (3.6 g/kg/d) administration for 8 weeks significantly reduced the swimming path length (*p* = 0.014) and escape latency (*p* = 0.006) of APP/PS1 mice. The escape latency (*p* = 0.006) and swimming path length (*p* = 0.012) in the PTP (1.8 g/kg) group were significantly reduced (Figure 3). Donepezil reduced both the swimming path length (*p* < 0.01) and escape latency (*p* < 0.01) significantly (Figures 3D,E). All treatment groups had no effect on the swimming speed (F = 0.279, *p* = 0.890) (Figure 3F).

### Effects of PTP on Neurotransmitters and Related Enzymes in the Brain

**FIGURE 4:**
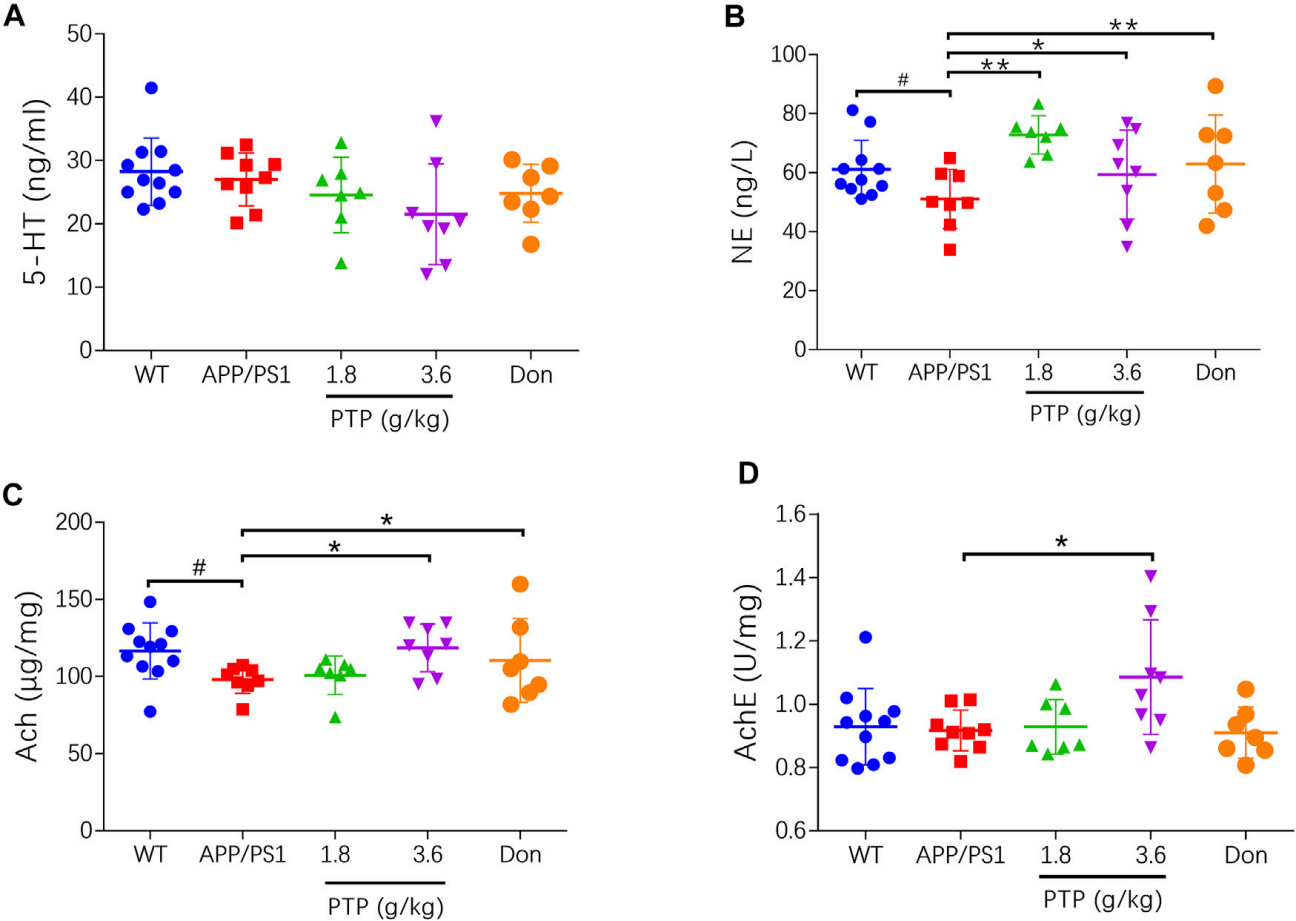
Effects of PTP on neurotransmitters and related enzyme in brain tissue. (**A)** 5-HT, **(B)** NE, **(C)** Ach, and **(D)** AchE. Compared with the wild-type group: ^#^ *p* < 0.05; compared with the APP/PS1 group: ^*^ *p* < 0.05, ^**^ *p* < 0.01. (*n* ≥ 7).

Compared with WT mice, APP/PS1 mice exhibited no significant changes in the brain 5-HT levels. PTP treatment for 8 weeks showed no effect on the 5-HT concentrations (*p* > 0.05) (Figure 4A). NE levels were decreased in APP/PS1 mice (*p* < 0.05), which were significantly improved by PTP and donepezil treatment (*p* < 0.05 or *p* < 0.01) (Figure 4B). Compared with that in the WT group, the Ach level in APP/PS1 mice was significantly decreased (*p* < 0.05), but AchE activity was not significantly changed (*p* = 0.801). After 8 weeks of treatment, the level of Ach and the activity of AchE in the brain tissue in the high-dose PTP group were significantly increased compared with those in the APP/PS1 group (*p* < 0.05). There were no significant changes in Ach levels (*p* = 0.622) or AchE activity (*p* = 0.766) in the low-dose PTP group. Donepezil significantly increased the Ach level (*p* < 0.05) but showed no effects on AchE activity (Figures 4C,D).

### Effects of PTP on Oxidative Stress in Brain Tissue

**FIGURE 5:**
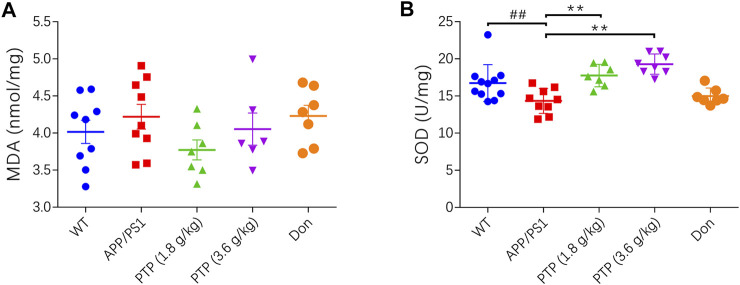
Effects of PTP on oxidative stress in brain tissue. (**A)** The effect of PTP on the concentration of MDA in brain tissue. **(B)** The effect of PTP on SOD activity in brain tissue. Compared with the wild-type group: ^##^ *p* < 0.01; compared with the APP/PS1 group: ^**^ *p* < 0.01. (*n* ≥ 7).

There were no differences between the WT group and the APP/PS1 group on the MDA level. After 8 weeks of treatment, there was no significant change in the MDA level in the positive control group or PTP group compared with the APP/PS1 group (F = 1.256, *p* = 0.307) (Figure 5A).

Compared with that in WT mice, the activity of SOD in APP/PS1 mice was significantly decreased (*p* < 0.01). After 8 weeks of treatment, compared with that in APP/PS1 mice, the activity of SOD in the PTP groups was significantly increased (*p* < 0.01), while there was no change in the donepezil group (*p* = 0.392) (Figure 5B).

### Effects of PTP on the Pathological Manifestations in the Hippocampal CA Region

**FIGURE 6:**
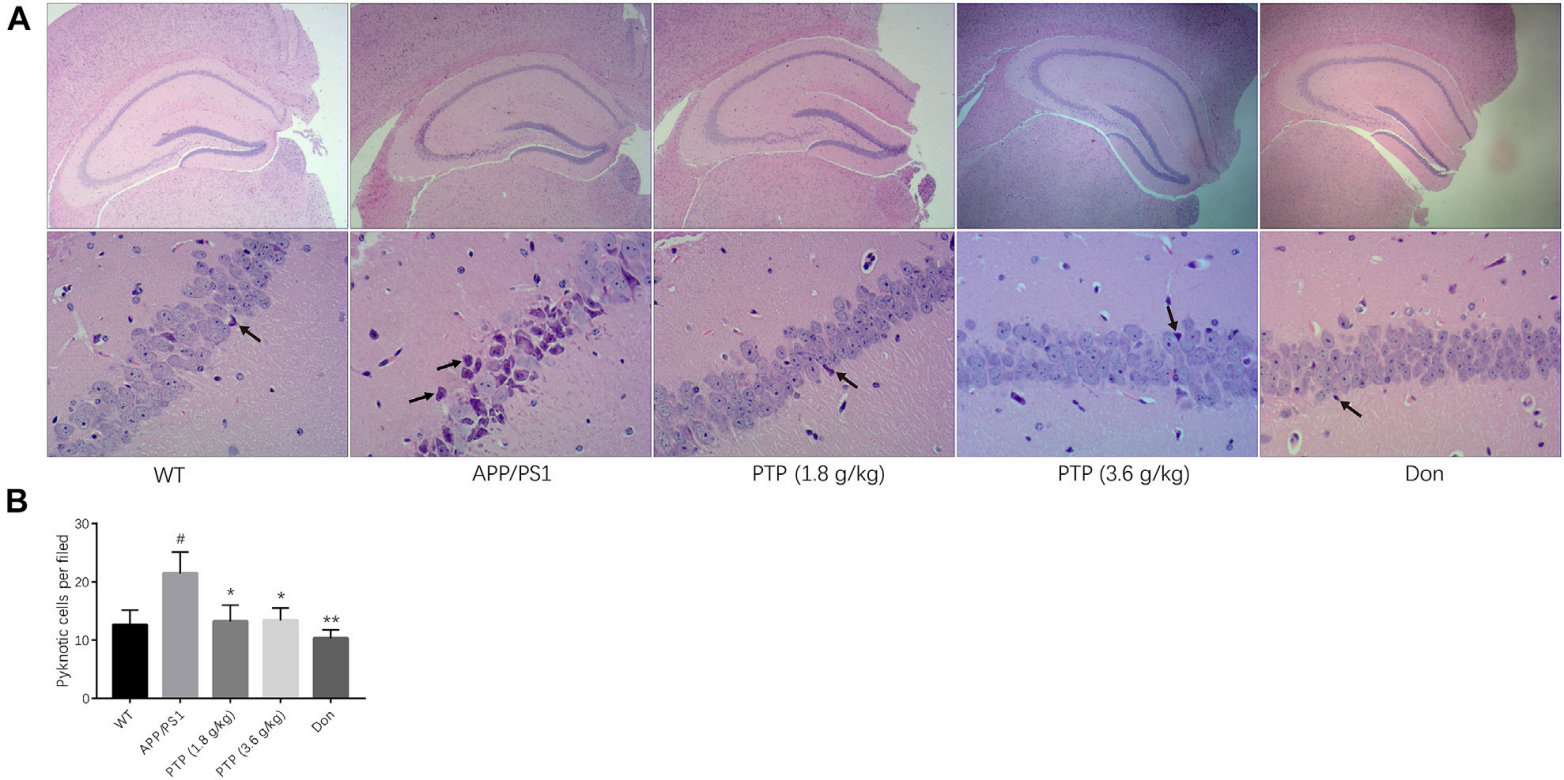
Evaluation of mice pathological manifestations in the hippocampal CA region. (**A)** Effects of PTP on pathological manifestations in the hippocampal CA region. The black arrow indicates pyknosis of the nerve cell nucleus. (HE staining, ×40 & ×200). **(B)** Quantitative analysis of the pyknotic cells in the hippocampal CA region. Compared with the wild-type group: ^#^ *p* < 0.05; compared with the APP/PS1 group: ^*^ *p* < 0.05, ^**^ *p* < 0.01. (*n* = 3).

Under a microscope, pyramidal cells in the hippocampal CA region of WT mice were densely arranged, with neat and plump cells. The nuclear membrane was smooth and uninterrupted, and the nucleoli were visible. In the APP/PS1 transgenic mouse group, pyramidal cells in the hippocampal CA region were loosely arranged. A small fraction of cells showed signs of neurodegeneration, such as wrinkled nuclei, hyperchromatic, and shrunken triangulated neuronal bodies. Some cells appeared structurally less intact, and a few cells were lost. Compared with those in the model group, the pyramidal cells in the donepezil and PTP groups were regularly arranged, the cytoplasmic and nuclear membranes appeared to be smooth and uninterrupted, and the nuclear pyknosis and deep staining were reduced (Figure 6A).

Quantitative analysis showed that the number of pyknotic cells per field was significantly higher in the APP/PS1 group than in the WT group (*p* < 0.05). PTP (1.8 g/kg and 3.6 g/kg) (*p* < 0.05) and donepezil (*p* < 0.01) treatment could rescue the cell pyknosis (Figure 6B).

### Effects of PTP on Autophagy and Apoptosis

**FIGURE 7:**
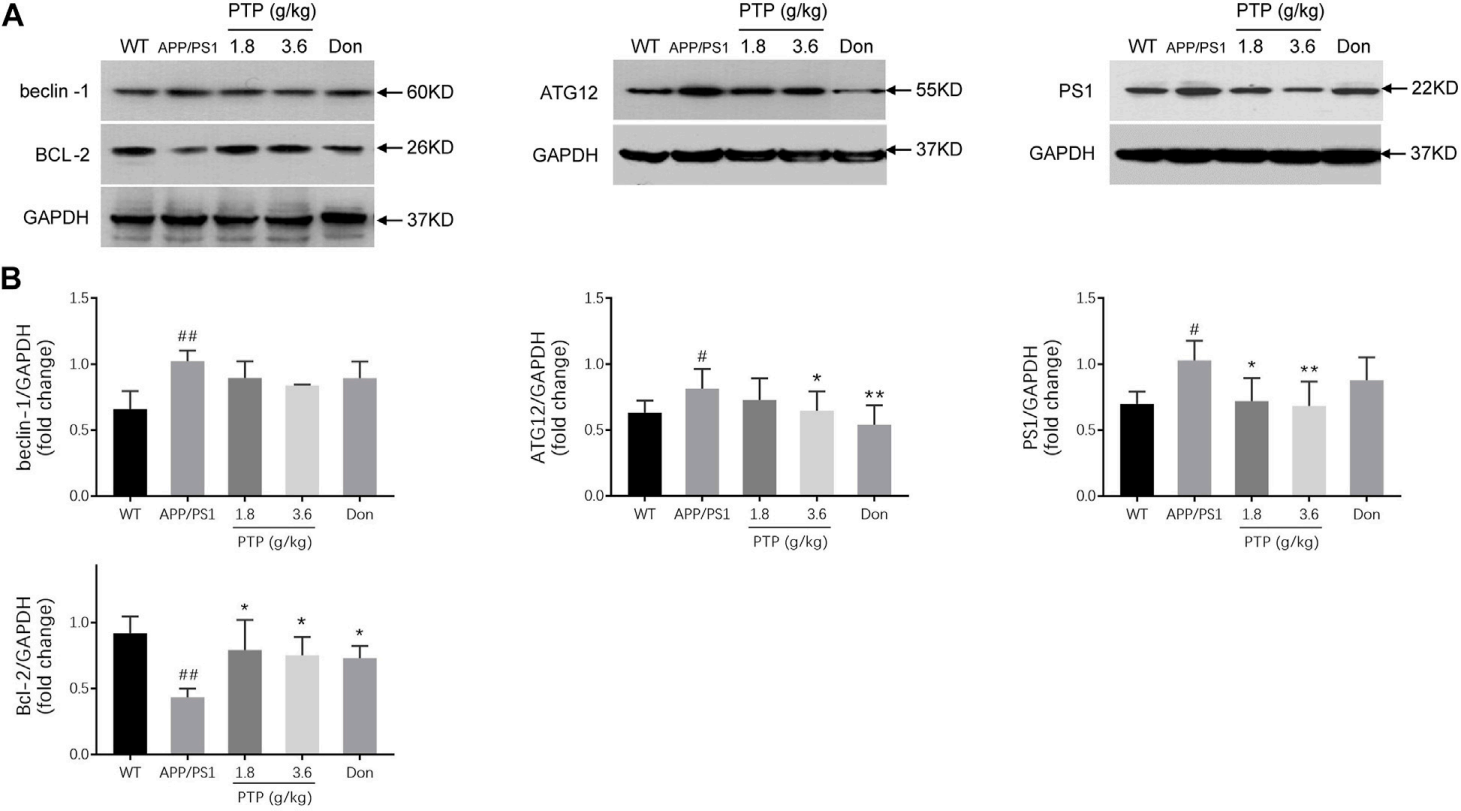
Effects of PTP on autophagy- and apoptosis-related proteins in the cerebral cortex **(A)** Western blotting was performed to detect the protein expression of beclin-1, Bcl-2, ATG12, and PS1. **(B)** The quantitative analysis of the protein expression of beclin-1, Bcl-2, ATG12, and PS1. Compared with the wild-type group: ^#^ *p* < 0.05, ^##^ *p* < 0.01; compared with the APP/PS1 group: ^*^ *p* < 0.05, ^**^ *p* < 0.01. (*n* = 3).

Compared with those in the WT group, the protein level of Bcl-2 was significantly decreased (*p* = 0.002), and the beclin-1 (*p* = 0.004), PS1 (*p* = 0.011), and ATG12 (*p* = 0.028) protein levels were significantly increased in the APP/PS1 transgenic mice. After 8 weeks of PTP (3.6 g/kg) treatment, the expression of Bcl-2 (*p =* 0.018) increased, and the expression of ATG12 (*p =* 0.027) and PS1 (*p =* 0.005) decreased significantly. PTP (1.8 g/kg) increased Bcl-2 (*p =* 0.049) expression, reduced PS1 (*p =* 0.011) expression, and whereas has no significant effect on ATG12 (*p =* 0.178) expression. Donepezil treatment increased the level of Bcl-2 (*p =* 0.027), reduced the expression of ATG12 (*p =* 0.002), whereas showed no significant effect on PS1 (*p =* 0.139) expression. The protein expression of beclin-1 decreased slightly in the PTP groups (PTP 3.6 g/kg, *p* = 0.111; PTP 1.8 g/kg, *p* = 0.202) and donepezil group (*p* = 0.209), but this reduction was not statistically significant (Figure 7).

## Discussion

This study showed that PTP could significantly improve the learning and memory abilities of APP/PS1 mice. The PTP groups showed increased neurotransmitters Ach and NE levels, reduced excessive autophagic activation, and suppressed oxidative stress and apoptotic activity.

Given the complexity of active components in PTP, it is difficult to characterize the pharmacological mechanisms of PTP for cognitive impairment by conventional methods. Therefore, we utilized a network pharmacological approach to analyze the active compounds and therapeutic targets of PTP. Having identified candidate targets, we further annotated their functions by GO and KEGG functional analysis with the DAVID database. This analysis highlighted that PTP might rescue cognitive impairment by several mechanisms, such as regulating apoptosis, autophagy, oxidative stress biological processes, and modulating apoptotic pathway, PI3K-Akt, signaling pathway, and MAPK signaling pathway. Since the number of GO and KEGG terms that meet the inclusion criteria is large and even up to several hundred, it is impossible to verify all the terms with pharmacology experiments. In the present study, we confirmed some of the terms related to the pathology of cognitive impairment as examples with animal experiments, including apoptosis, autophagy, and oxidative stress.

The APP/PS1 transgenic mouse model was used in this study. APP/PS1 transgenic mice express a mutated fusion of human presenilin (DeltaE9) and human amyloid precursor protein (AppSwe), leading to cognitive impairment. APP/PS1 transgenic mice showed cognitive-behavioral changes at 3 months of age, plaques at 5 months of age, and a large number of plaques at 12 months of age. In APP/PS1 transgenic mice, the distribution of Aβ protein deposition is largely similar to that of human AD and senile cognitive impairment. APP/PS1 transgenic mouse model can be used to simulate cognitive impairment in AD (Zha et al., 2020).

The Morris water maze is an important tool for testing the memory abilities of animals (Mifflin et al., 2021). The classic Morris water maze test evaluates the ability of mice trained to find a hidden platform in a fixed position and achieve stable spatial recognition. This kind of memory is a type of spatial reference memory, and its storage location mainly involves the limbic system, and related cerebral cortex regions. In this study, APP/PS1 transgenic mice were used as models, and the swimming duration and swimming path length of mice in the water maze within 2 min were used as the main indexes to observe the effects of PTP on the swimming performance of the mice. The results showed that after treatment with PTP for 8 weeks, the swimming performance of APP/PS1 transgenic mice in the water maze was significantly improved, suggesting that PTP could improve the spatial learning and memory abilities of APP/PS1 mice. The escape latency and distance were evoked on day 4 in the APP/PS1 group, which is different from the other groups. Researchers still do not have a clear explanation on the candidate reasons for this issue, although a similar phenomenon has also been reported by several other studies (D'Agostino et al., 2013; Lu et al., 2014; Zhou et al., 2016; Banks et al., 2018). Theoretically and empirically, it might be related to the pathological processes of cognitive impairment and the specific characteristics of the APP/PS1 transgenic mouse model.

Brain monoamine neurotransmitters are closely related to mental activity, emotion, behavior, and body movement, and there is also a certain correlation with learning and memory (Bekdash, 2021). Abnormal NE and 5-HT levels can lead to decreased brain excitability, resulting in memory loss and depressive symptoms. The lesions associated with AD often involve the locus ceruleus and dorsal raphe nucleus and are characterized by abnormal levels of NE and 5-HT in the corresponding brain regions and decreased learning and memory abilities (Trillo et al., 2013; Šimić et al., 2017). Modulating the levels of monoamine transmitters in the brain may help improve learning and memory abilities. In this study, APP/PS1 transgenic mice showed no reduction in 5-HT levels, and PTP failed to regulate the 5-HT level, which still needs to be further explored. APP/PS1 mice showed significantly decreased NE levels, and after 8 weeks of PTP administration, the level of NE in the brain tissue increased significantly, suggesting that PTP may play a role in regulating certain monoamine neurotransmitters. The NE level of the PTP (1.8 g/kg) group increased more than that of the PTP (3.6 g/kg) group. Such phenomenon shown in this study was probably due to the complex composition of PTP. Unlike the pharmacological characterization of an individual compound, complex compound preparations often do not show a dose-effect relationship and sometimes even show reversed dose-effect relationships. Results showed that donepezil also evokes the NE level. NE and Ach belong to different neurotransmitter systems. Although donepezil is well known as the AchE inhibitor, evidence from animals (Sakr et al., 2014; Kaundal et al., 2018), and humans (Ma et al., 2020) showed donepezil could also enhance the NE level in cognitive impairments. However, the underlying mechanisms have not yet been fully elucidated.

The brain cholinergic system is closely related to cognitive function and is usually damaged in AD patients (Cheng et al., 2021). As an essential transmitter in the human and mammalian brain, Ach is involved in the physiological and pathological processes of learning and memory, sleep and wake, as well as a variety of nervous system diseases, such as AD, and Parkinson’s disease. One of the main clinical manifestations of AD is decreased Ach synthesis in the brain. The Ach concentration becomes an important parameter to measure learning and memory abilities (Dineley et al., 2015). A change in the Ach level before and after medication can reflect the effect of drugs on the cholinergic system more intuitively than AchE activity. The low levels of Ach in the brains of APP/PS1 transgenic mice were similar to those seen in AD patients. The prominent pathological manifestations of AD are abnormalities in the cholinergic system, including AchE hyperactivity and decreased Ach levels. The administration of an AchE inhibitor is an effective method to increase the level of Ach in the brains of patients. In this study, compared with WT mice, APP/PS1 transgenic mice showed significant decreases in brain Ach levels and no significant changes in AchE activity. The results might indicate the reduced brain Ach synthesis but not the enhancement of brain Ach degradation, which led to a decrease in cholinergic system function. This finding is consistent with the reduction in Ach synthesis in AD patients. After 8 weeks of PTP administration, the levels of Ach and the activity of AchE in the brains of the APP/PS1 transgenic mice were significantly increased, suggesting that PTP may increase the level of Ach by increasing its synthesis. It implies that PTP may improve the abnormal function of the brain cholinergic system in AD by regulating the abnormal metabolic state of Ach, thus improving the learning and memory abilities. Although it seems unreasonable that the AchE activity didn’t change in the donepezil treatment group, previous studies of the effect of donepezil on AchE activity in APP/PS1 showed controversial results (Ye et al., 2015; Shi et al., 2018). As shown in the previous study (Abe et al., 2003), the maximal AchE inhibition of donepezil occurs at about 1 hour after drug administration in mice, and the inhibition diminishes with time passing. The above finding of the inhibitory effect of donepezil on AchE may be associated with its pharmacodynamic characteristics, as donepezil was reported to have a half-life of about 3 hours in rats (Goh et al., 2011; Nirogi et al., 2012). The results showed that PTP modulated the function of the brain cholinergic system via a mechanism distinct from that of donepezil which maintains Ach levels by inhibiting Ach degradation rather than increasing Ach synthesis, whereas this effect has been shown to be time-limited in clinical practice. The high dose PTP increased the AchE activity as well as Ach level. Since PTP is a complex compound preparation, it might have the effect of increasing both the concentrations of Ach and AchE, and the elevated AchE was not sufficient to completely degrade the increased Ach.

The toxic effects of oxidative stress and free radical damage on nerve cells have been well established (Mattson and Arumugam, 2018). Previous studies have shown that oxidative stress and free radical damage significantly increase with aging (Cochemé et al., 2011; Upadhyay et al., 2020). The nervous system is particularly sensitive to free radical damage due to its rich lipid composition, relative lack of antioxidant enzymes, and high oxygen consumption. Mitochondrial function declines during aging, and excessive free radicals accumulate, attacking the body, causing oxidative stress, resulting in the peroxidation of lipids, proteins, and DNA and eventually leading to nerve cell death. SOD and MDA are two classical indexes reflecting systemic redox homeostasis. SOD is the most important antioxidant enzyme, and MDA is an oxidative product produced when reactive oxygen species attack fat tissue. The level of MDA can reflect the degree of lipid peroxidation and reflect the degree of cell damage better than reactive oxygen species. The activity of SOD in the brains of APP/PS1 transgenic mice was significantly decreased, indicating decreased antioxidant capacity, and which was consistent with the clinical lesions of aging and senile patients with cognitive impairment. After the administration of PTP, the activity of SOD in brain tissue was significantly increased. The results showed an opposite trend for the MDA and SOD results, although some of the results did not show significant differences, indicating that PTP may significantly improve free radical scavenging in the brain, which may be helpful in anti-aging by preventing or delaying age-related pathological processes.

The integrity of the brain tissue structure is the basis of normal function. Physical or pathological factors, such as aging and AD, lead to morphological and functional abnormalities of the brain tissue. The hippocampal CA region is a common brain region affected by aging and AD (Zeier et al., 2011). In this study, APP/PS1 transgenic mice exhibited neuronal loss, apoptosis, and disordered arrangement in the hippocampal CA area, indicating functional impairment of the corresponding region. PTP and donepezil rescued the apoptosis and neuron loss, suggesting their protective effects on cognitive impairment.

Autophagy is a lysosomal-dependent physiological process that helps the body remove and degrade damaged and denatured proteins, aging and dysfunctional cells and organelles to maintain the balance of protein metabolism and the stability of the intracellular environment (Cai et al., 2016). The autophagic activity of APP/PS1 mice is various at different age. At 4–5 months old, the autophagy process is initiated in the brain tissue; at 6–8 months old, the autophagic activity was excessively activated; at 10 months old, large amounts of autophagic vacuoles and lysosome were observed in the brain of APP/PS1 mice. The formation of autophagosomes is a complex biological process. Beclin-1 and ATG12 are involved in the formation of autophagosomes. Once the autophagosome fuses with the lysosome, the autophagosome can be degraded by the lysosome. This study showed that compared with that in WT mice, the protein expression of beclin-1 and ATG12 in APP/PS1 transgenic mice was significantly increased, which indicated that autophagic activity of APP/PS1 transgenic mice was excessively increased and reflected the increased pathology severity indirectly. After 8 weeks of PTP administration, the protein expression of beclin-1 and ATG12 decreased. Although it is unclear the cause-and-effect relationship between the effect of PTP on autophagy and the other pathology changes, the results suggest that PTP may effectively alleviate the pathology severity of the brain tissue, and suppress the hyperactivated autophagy levels in the brain. Furthermore, since the autophagy flow is the primary indicator in determining the autophagy activity, which is a dynamic process, further research on the effect of PTP in regulating autophagy flow-related proteins is needed. For the issue of donepezil’s effect on ATG12, no relevant literature has been reported so far. The effect of donepezil on autophagy is rarely reported in APP/PS1 mouse model. A rat model of vascular dementia showed that donepezil could inhibit autophagy, whereas evidence from a cardiac ischemia/reperfusion injury gave opposite conclusions (Jiang et al., 2019; Khuanjing et al., 2021). Thus, the effect and mechanism of donepezil on autophagy still require further investigation.

Apoptosis is one of the main pathogeneses of neurodegenerative diseases. A decrease in apoptosis is related to the stability of cognitive function (Friedlander, 2003; Wingo et al., 2019). Inhibiting brain cell apoptosis may be beneficial in ameliorating age-related cognitive decline (Ramos-Miguel et al., 2017). Bcl-2 is an anti-apoptotic protein that can significantly inhibit apoptosis. Abnormal PS1 can induce neuronal apoptosis, resulting in neuronal loss. This study showed that the expression of Bcl-2 in APP/PS1 mice was decreased, and the expression of PS1 was increased, suggesting that apoptosis was up-regulated in brain cells. PTP up-regulated the expression of the anti-apoptotic protein Bcl-2 and downregulated the expression of the apoptosis-related proteins PS1 in APP/PS1 mice, suggesting that PTP could inhibit neuronal apoptosis in the brain. Multiple studies have documented donepezil could enhance the Bcl-2 level (Takada-Takatori et al., 2006; He et al., 2018; Zaitone et al., 2020). The possible mechanism may be that donepezil inhibited apoptosis by activating the PI3K/Akt/Bcl-2 pathway (Zaitone et al., 2020). Donepezil affords excess Ach, which binds to α7 nicotinic acetylcholine receptor and then activates its channels, permitting sodium and calcium influx. Then, calcium binds to calcium reliant enzymatic reactions that elicit the PI3K/Akt/Bcl-2 pathway.

In this study, the existing positive results could support the conclusion effectively, including the efficacy indicators, such as behavioral parameters, neurotransmitters, and HE staining result, and those related to the key mechanisms, including autophagy oxidative stress, and apoptosis. However, this study also had some limitations. Firstly, Aβ accumulation is one of the most important hypothetical pathologies for AD, and there are several key fragments of Aβ peptides concerning AD pathology hypothetically. However, from an alternative perspective, although Aβ has been the prime suspect for driving pathology in AD, its effect on AD treatment is still controversial. Recently, a meta-analysis showed anti-Aβ interventions are unlikely to have an important impact on slowing cognitive or functional decline (Lu et al., 2020). Basic research also indicates that these proteins have been highly conserved throughout evolution and may have crucial physiological roles, and Aβ-targeting therapies may unintentionally disrupt such functions. A balanced consideration of both the physiological and pathological roles of Aβ will be essential for designing safe and effective therapeutics (Kent et al., 2020). This study investigated the effect of PTP on PS1 protein but did not further verify whether PTP regulated the production of APP and Aβ or tau protein phosphorylation. Future studies still need to examine the regulatory effect of PTP on biological markers related to Aβ and its mechanism. Secondly, autophagy is another critical mechanism of AD. We found the autophagy-related protein ATG12 was down-regulated in the PTP group. However, since the autophagy flow is the primary indicator in determining the autophagy activity, which is a dynamic process, and further research on the effect of PTP in regulating autophagy flow-related proteins is needed.

## Conclusion

The present work showed that PTP could improve the cognitive function of APP/PS1 mice. The mechanism may be related to the improvement of neurotransmitter Ach and NE levels; the reduction of the excessive autophagic activation, which manifests as ATG12 decrease; the suppression of oxidative stress by increasing SOD level; and inhibiting apoptosis process by up-regulating Bcl-2 and down-regulating PS1.

The present study is the first systematic research of the effect of PTP in treating cognitive impairment by network pharmacology approach and verified by conducting a wide range of animal experiments. This work effectively confirmed the effect and mechanisms of PTP on cognitive impairment and provided an exemplar for similar studies in pharmacological research of traditional Chinese medicine compound preparations.

Despite these important discoveries, this study has limitations. The Aβ accumulation and tau protein phosphorylation are important hypothetical pathologies for AD. However, the effect of PTP on the production of Aβ or tau protein phosphorylation was not verified in the present study, which needs to be further explored in the future. Autophagy is another critical mechanism of AD, and the autophagy flow is the primary indicator in determining autophagy activity. We only observed the effect of PTP on some particular autophagy-related proteins but did not verify the effect of PTP on the whole autophagy flow. Thus, further research on the effect of PTP in regulating more autophagy flow-related proteins is needed.

The effect of PTP on cognitive impairment has been confirmed in clinical and basic studies. However, the composition of PTP is complex. How to simplify its components to obtain effective and relatively low-cost alternative formulas or compound preparations will be the focus of future work.

## Data Availability

The original contributions presented in the study are included in the article/Supplementary Material, further inquiries can be directed to the corresponding authors.
