# Supplementary material for: Pantao Pill Improves the Learning and Memory Abilities of APP/PS1 Mice by Multiple Mechanisms
Source: Front Pharmacol. 2022 Feb 25;13:729605. doi: 10.3389/fphar.2022.729605 (PMC8915116; doi:10.3389/fphar.2022.729605)
Supplement: Supplementary file 3 [file DataSheet2.docx]

# *Supplementary Material S2*


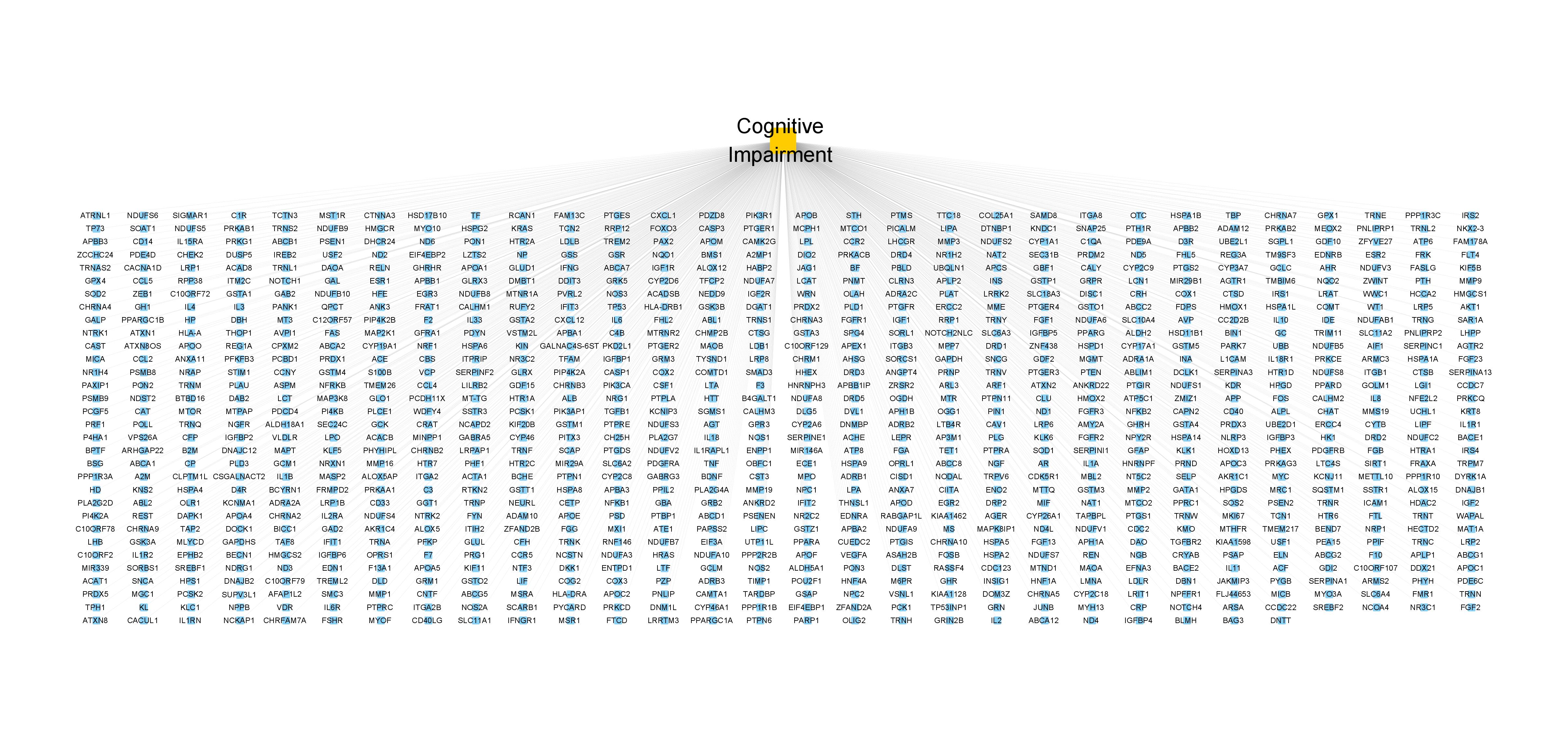


**Supplementary Figure S2**. Cognitive impairment target network.

The yellow node represents cognitive impairment, and the blue nodes represent targets related to cognitive impairment.


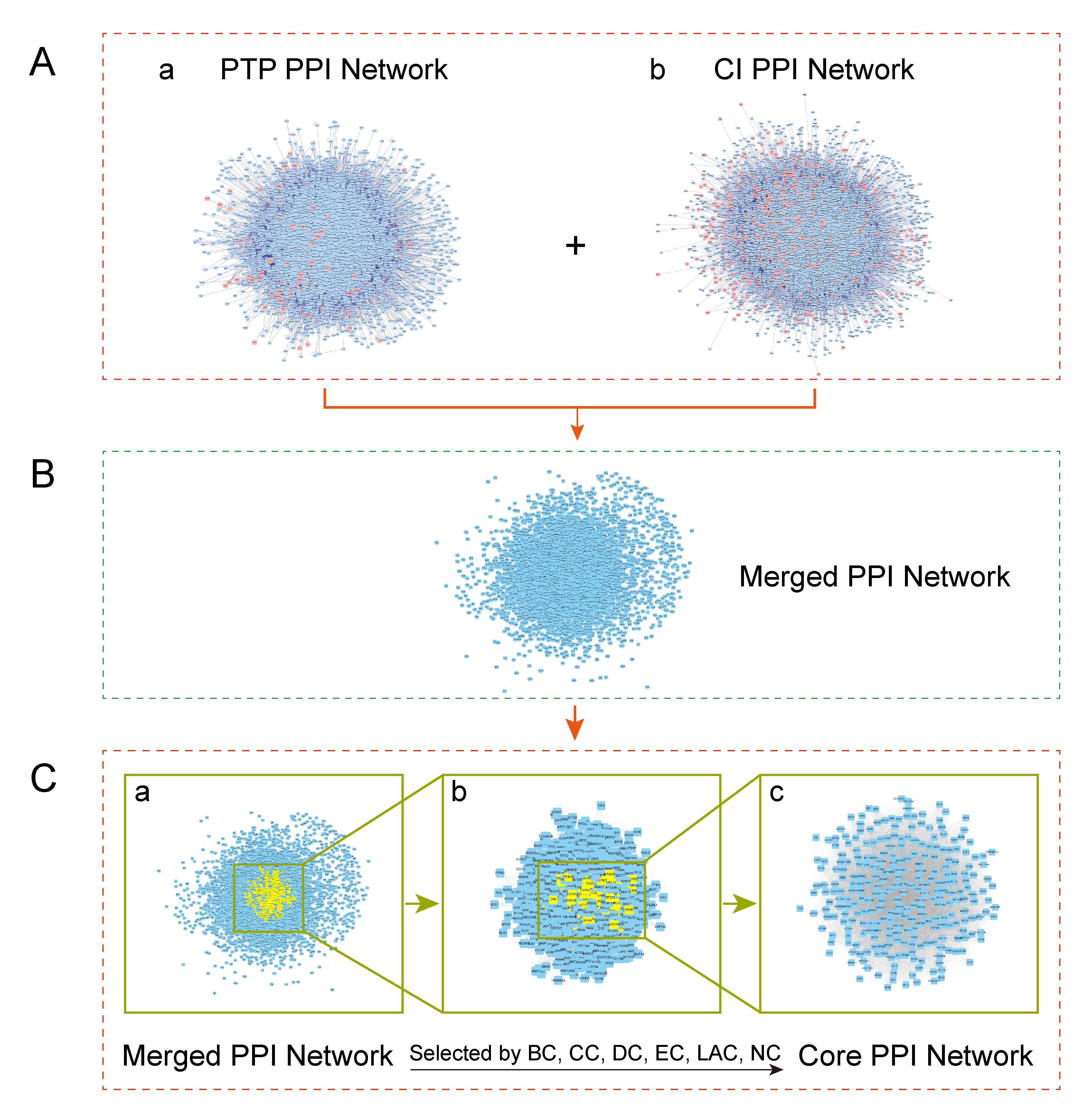


**Supplementary Figure S3**. Construction of the core PPI network.

**(A)** PTP and cognitive impairment (CI) PPI networks. **(B)** Intersection network of the PTP PPI network and CI PPI network. **(C)** Topology analysis to screen the core network. The first screening was performed with a boundary that is greater than twice the median of the DC; DC, BC, CC, EC, LAC, and NC were used as references for the second screening; the core PPI network was obtained after two screenings.
