# Supplementary material for: Pantao Pill Improves the Learning and Memory Abilities of APP/PS1 Mice by Multiple Mechanisms
Source: Front Pharmacol. 2022 Feb 25;13:729605. doi: 10.3389/fphar.2022.729605 (PMC8915116; doi:10.3389/fphar.2022.729605)
Supplement: Supplementary file 5 [file DataSheet1.docx]

# *Supplementary Material S1*

# UPLC- LTQ-Orbitrap Fingerprint Map Establishment

# 1 Materials and Methods

# 1.1 Materials

Pantao pill (PTP) refined powder (batch number P004) was provided by Darentang Pharmaceutical Factory (Tianjin, China). The preparation process of PTP is as below: the raw materials of the traditional Chinese medicines are mixed, dried and crushed into fine powder, which was then sieved and sterilized to be the refined powder for the pill preparation of PTP. Ginsenoside Rg3 and other reference products were purchased from the Institute for Biological Products Control, National Institutes for Food and Drug Control and Chengdu Purify Technology Development Co., Ltd. The structures of the reference products were determined by ^1^H-NMR and ^13^C-NMR, and the purity was measured by the HPLC normalization method, all of which were greater than 95%, indicating that these substances could be used for qualitative research.

Chromatographic quality acetonitrile and methanol were purchased from Fisher Scientific (Fair Lawn, NJ, USA), analytical-grade methanol and formic acid were purchased from Sigma Aldrich (St. Louis, Mo, USA), ultrapure water was prepared by the Millipore Synergy UV Ultrapure Water mechanism. The 0.22 µm microporous filter membrane was purchased from Beijing Huazhi Chromatography Technology Co., Ltd.

# 1.2 Sample Preparation

Preparation of the test product solution was performed by taking 0.3 g of PTP coarse powder (i.e., intermediate without auxiliary materials) and placing it in a 50 mL erlenmeyer flask, adding 25 mL of methanol, weighing the solution, and ultrasonicating it at room temperature (250 W, 70 kHz) for 30 min. Then, the powder was cooled to room temperature and weighed again, and methanol was added to make up the lost weight, after which the solution was filtrated with a 0.22 μm microporous membrane, and the filtrate was collected.

To prepare the standard solution, an appropriate amount of reference substance was collected, and methanol was added to make a solution containing approximately 50 μg of each reference substance per milliliter, which was stored at 4°C and kept away from light.

# 1.3 Experimental Conditions

UPLC Conditions: Waters ACQUITY UPLC HSS T3 (2.1×100 mm, 1.8 μm) chromatographic columns were used for LC-MS analysis. Gradient elution was performed with 0.1% formic acid as mobile phase A and acetonitrile as mobile phase B at room temperature. The injection volume was 3 μL, and the flow rate was 0.3 mL/min.

The elution gradient was: 0-2 min, 8% B; 2-20 min, 8-26% B; 20-22 min, 26% B; 22-30 min, 26-42% B; 30-50 min, 42-60% B; 50-55 min, 60-95% B; 55-58 min, 95% B, 58-60 min, 95-8% B; and 60-65 min, 8% B.

MS Conditions: In negative ion mode, the capillary temperature was 350℃, the sheath gas flow rate (nitrogen) was 30 arb, the auxiliary gas flow rate (nitrogen) was 10 arb, the spray voltage was 3 kV, the capillary voltage was −35 V, and the tube lens voltage was −110 V. The mass axis accuracy of high-resolution mass spectrometry was corrected with mixed standard solutions of caffeine, sodium dodecyl sulfate, sodium taurocholate, tetrapeptide MRFA and Ultramark, and the mass accuracy error was within 5 PPM. The quality detection range was *m/z* 100−1200. The sample was scanned by a full scan with high-resolution FT (full scan (FS)). The R resolution was set as 30000. An ion trap electrode was used to detect ion fragments. Dynamic exclusion (DE) was enabled and the repeat count was set to 3, the repeat duration was set to 10 s, the exclusion list size was set to 100, and the exclusion duration was set to 20 s.

# 2 Results

According to the above conditions, total ion flow diagrams of PTP were obtained (**Supplementary Figure S1**). According to the literature reports and combined with the chromatographic retention behavior, molecular weight information and mass spectrometry fragmentation rule, the chemical constituents in PTP were preliminary identified. Results showed the main components of PTP included flavonoids, triterpenoid saponins, steroid saponins, organic acids, iridoid glycosides, and phenylethanol glycosides.

Moreover, combining literature reports and related reference materials collected by our research group in the early stage, each chromatographic peak in the MS map was identified (**Supplementary Table S1**).


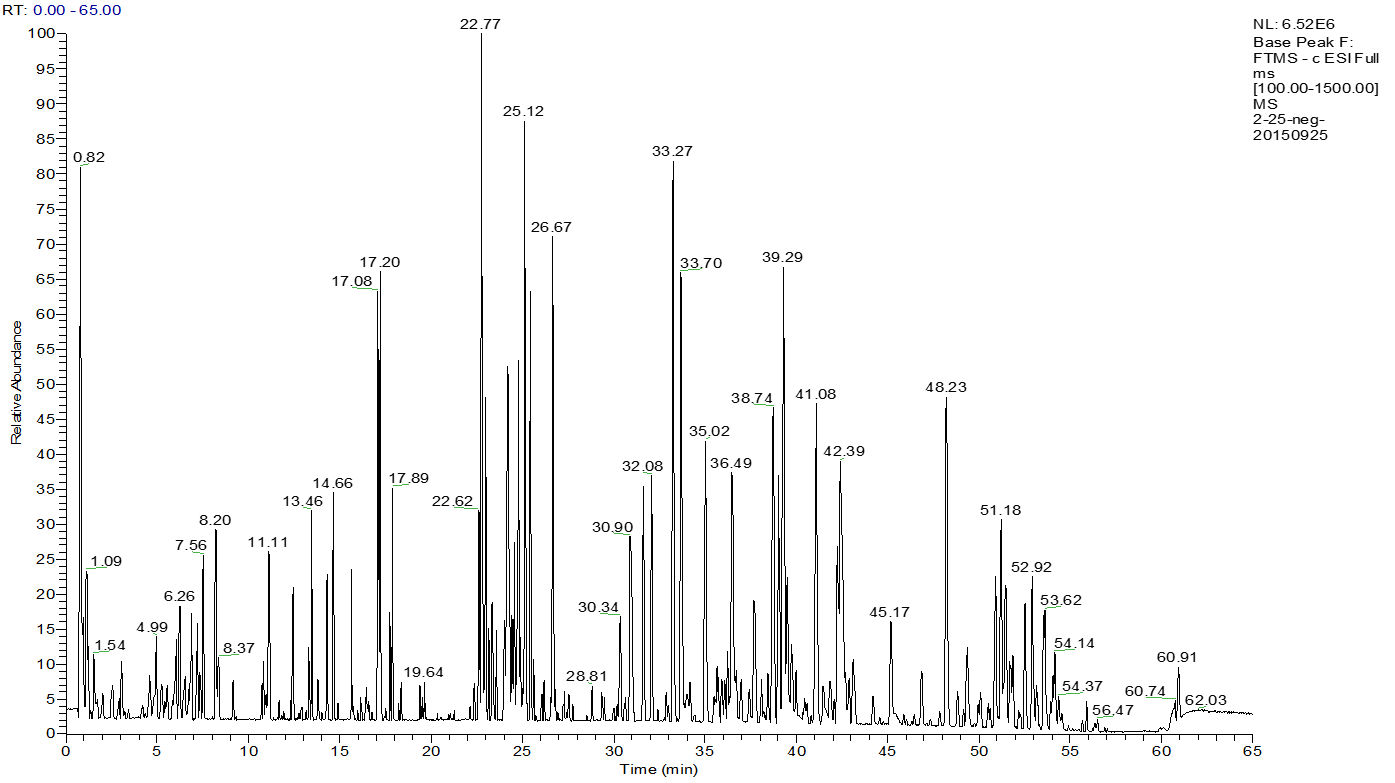


**Supplementary Figure S1.** UPLC-LTQ-Orbitrap high-resolution mass spectrometry total ion chromatogram of PTP.

**Supplementary Table S1.** Structural identification of chemical components in PTP preparation based on UPLC-LTQ-Orbitrap HRMS

| Peak | t_R_  /min | Formula | Theoretical Mass/*m/z* | Experimental  Mass/*m/z* | Error  /ppm | ESI–MS/MS (*m/z*) | Structural identification |
| --- | --- | --- | --- | --- | --- | --- | --- |
| 1 | 0.79 | C_11_H_16_NO_8_ | 290.0881 | 290.0879 | -0.69 | 200(100),272(20),254(12) | Pyroglutamic acid hexose |
| 2 | 1.27 | C_22_H_27_O_14_ | 515.1395 | 515.1391 | -0.78 | 353(100),191(84),191(81),179(57),395(23) | Glucoside 3-caffeoylquinic acid |
| 3 | 1.81 | C_22_H_27_O_14_ | 515.1395 | 515.1391 | -0.78 | 323(100),191(32),353(24),173(21),191(16) | 4-glucoside of caffeic acid quinic acid |
| 4 | 1.96 | C_22_H_27_O_14_ | 515.1395 | 515.1386 | -1.75 | 353(100),191(76),179(50),373(30),135(13) | 4-glucoside of caffeic acid quinic acid |
| 5 | 2.26 | C_9_H_7_O_4_ | 179.0339 | 179.0344 | 2.79 | 135(100),161(2) | Caffeic acid isomer |
| 6^Δ^ | 2.49 | C_16_H_17_O_9_ | 353.0867 | 353.0874 | 1.98 | 191(100),179(51) | Green chlorogenic acid |
| 7 | 2.55 | C_16_H_23_O_10_ | 375.1286 | 375.1295 | 2.40 | 213(100),169(52),357(12),151(10) | 8-Epistrychnic Acid |
| 8 | 2.75 | C_21_H_27_O_13_ | 487.1446 | 487.1478 | 2.31 | 229(100),323(96),325(74),163(62) | Cistanche F |
| 9 | 3.44 | C_16_H_19_O_9_ | 355.1029 | 355.1034 | 1.41 | 193(100),134(2),178(2) | Ferulic acid glucoside |
| 10 | 4.04 | C_9_H_10_O_4_N | 196.0615 | 196.0617 | 1.02 | 124(100),122(67),152(48),94(5) | N-hydroxy-L-tyrosine |
| 11 | 4.28 | C_9_H_7_O_4_ | 179.0350 | 179.0351 | 0.56 | 135(100),161(12) | Caffeic acid isomer |
| 12^Δ^ | 4.53 | C_16_H_17_O_9_ | 353.0867 | 353.0871 | 1.13 | 191(100),179(12) | Chlorogenic acid |
| 13 | 5.08 | C_15_H_17_O_8_ | 325.0929 | 325.0928 | -0.31 | 163(100),119(7),311(1) | Coumaric acid hexose |
| 14^Δ^ | 5.42 | C_16_H_17_O_9_ | 353.0867 | 353.0877 | 2.83 | 173(100),179(36),191(18) | Cryptochlorogenic acid |
| 15 | 5.85 | C_33_H_39_O_21_ | 771.1984 | 771.1990 | 0.78 | 609(100),301(6),463(2) | Rutin glucose |
| 16^Δ^ | 5.89 | C_9_H_7_O_4_ | 179.0339 | 179.0344 | 2.79 | 135(100),161(35) | Caffeic acid |
| 17 | 6.20 | C_39_H_49_O_26_ | 933.2517 | 933.2521 | 0.43 | 609(100),301(21),300(7) | Rutin diglucoside |
| 18 | 6.57 | C_10_H_9_O_4_ | 193.0495 | 193.0505 | 5.18 | 149(100),178(73),134(21) | Ferulic acid |
| 19 | 6.63 | C_39_H_49_O_26_ | 933.2517 | 933.2520 | 0.32 | 609(100),301(7),625(3),300(2) | Rutin diglucoside isomer |
| 20 | 7.05 | C_16_H_17_O_8_ | 337.0928 | 337.0926 | -0.59 | 163(100),191(19),173(15),127(9) | 3-cinnamyl quinic acid |
| 21 | 7.49 | C_16_H_17_O_8_ | 337.0928 | 337.0926 | -0.59 | 191(100),163(27),127(16),173(13) | 5-cinnamyl quinic acid |
| 22 | 7.90 | C_27_H_29_O_15_ | 593.1532 | 593.1515 | -2.87 | 473(100),353(47),503(35),383(24) | Vitexin New Zealand Ⅱ |
| 23 | 8.21 | C_17_H_19_O_9_ | 367.1024 | 367.1026 | 0.54 | 193(100),173(15),134(10),178(4) | 3-feruylquinic acid |
| 24 | 8.39 | C_17_H_19_O_9_ | 367.1024 | 367.1021 | -0.82 | 191(100),193(35),127(20),173(16) | 5-feruylquinic acid |
| 25 | 9.19 | C_35_H_45_O_20_ | 785.2499 | 785.2509 | 1.27 | 623(100),461(6) | Echinoside isomer |
| 26 | 9.63 | C_35_H_45_O_20_ | 785.2499 | 785.2512 | 1.66 | 623(100),461(9) | Echinoside isomer |
| 27^Δ^ | 9.71 | C_35_H_45_O_20_ | 785.2499 | 785.2510 | 1.40 | 623(100),461(12) | Echinacoside |
| 28 | 10.30 | C_27_H_29_O_15_ | 593.1512 | 593.1513 | 0.17 | 413(100),293(43),473(8),341(3) | Vitexin glucoside isomer |
| 29 | 10.30 | C_27_H_29_O_15_ | 593.1532 | 593.1513 | -3.20 | 413(100),293(43),473(8) | Vitexin glucoside |
| 30 | 10.92 | C_28_H_31_O_15_ | 607.1685 | 607.1671 | -2.31 | 487(100),427(95),445(74),325(24),307(15) | Spinosin |
| 31 | 10.94 | C_13_H_13_O_3_N_2_ | 245.0932 | 245.0931 | -0.41 | 203(100),116(5),201(3) | N-Acetyl-DL-tryptophan |
| 32 | 11.01 | C_28_H_31_O_15_ | 607.1685 | 607.1669 | -2.64 | 427(100),307(10),487(8),445(7) | Isospinol |
| 33^Δ^ | 11.06 | C_27_H_29_O_16_ | 609.1450 | 609.1445 | -0.82 | 301(100),447(28),179(15),151(12),193(7) | Rutin |
| 34^Δ^ | 11.31 | C_21_H_19_O_12_ | 463.0871 | 463.0887 | 3.46 | 301(100),300(23),178(21),151(14) | Quercetin |
| 35 | 11.58 | C_22_H_21_O_10_ | 445.1129 | 445.1143 | 3.15 | 325(100),297(58),231(24),282(21),216(14) | Swertisin |
| 36^Δ^ | 11.67 | C_21_H_19_O_12_ | 463.0871 | 463.0877 | 1.30 | 301(100),178(27),151(21) | Isoquercitrin |
| 37 | 11.73 | C_18_H_18_O_4_N | 312.1236 | 312.1241 | 1.60 | 178(100),297(68),135(41) | Lycium barbarum methylamide/isomer |
| 38^Δ^ | 12.34 | C_29_H_35_O_15_ | 623.1970 | 623.1976 | 0.96 | 461(100),315(2) | Acteoside |
| 39 | 12.68 | C_27_H_29_O_15_ | 593.1529 | 593.1508 | -3.54 | 285(100),284(5),257(5) | Nicotiflorin |
| 40 | 12.74 | C_37_H_49_O_20_ | 813.2812 | 813.2826 | 1.72 | 637(100),619(25),491(6),473(6) | Jionoside B |
| 41 | 12.96 | C_25_H_23_O_12_ | 515.1184 | 515.1179 | -0.97 | 353(100),393(92),191(69),179(73) | 1,3-DiCQA |
| 42 | 13.02 | C_28_H_31_O_16_ | 623.1612 | 623.1620 | 1.28 | 315(100),300(20),271(9) | Isorhamnetin 3-O-rutinoside |
| 43^Δ^ | 13.24 | C_29_H_35_O_15_ | 623.1970 | 623.1979 | 1.44 | 461(100),315(1) | Isoacteoside |
| 44^Δ^ | 13.29 | C_25_H_23_O_12_ | 515.1184 | 515.1177 | -1.36 | 353(100),173(21),335(17) | 3, 4-DiCQA |
| 45^Δ^ | 14.09 | C_25_H_23_O_12_ | 515.1184 | 515.1176 | -1.55 | 353(100).191(17),179(14),135(10) | 3, 5-DiCQA |
| 46 | 14.52 | C_25_H_23_O_12_ | 515.1184 | 515.1177 | -1.36 | 353(100),191(90),179(28),135(18),434(5) | 1, 5-DiCQA |
| 47^Δ*^ | 15.95 | C_48_H_81_O_20_ | 977.5316 | 977.5342 | 2.66 | 931(100),799(27) | Panax notoginseng saponin R_1_ |
| 48 | 16.29 | C_18_H_18_O_4_N | 312.1236 | 312.1238 | 0.64 | 178(100),297(89),135(65) | Lyciumide A isomer |
| 49^Δ^ | 16.83 | C_43_H_73_O_16_ | 845.4893 | 845.4884 | -1.06 | 799(100),637(49),475(20) | Ginsenoside Rg_1_ |
| 50^Δ*^ | 16.95 | C_49_H_83_O_20_ | 991.5472 | 991.5487 | 1.51 | 945(100) | Ginsenoside Re |
| 51 | 21.48 | C_10_H_9_O_4_ | 193.0495 | 193.0508 | 6.73 | 193(100),109(49),108(40),178(7),149(6) | Ferulic acid/isomer |
| 52* | 22.40 | C_43_H_73_O_16_ | 845.4893 | 845.4898 | 0.59 | 799(100),475(64),637(83) | Ginsenoside Rf |
| 53 | 22.60 | C_50_H_81_O_22_ | 1033.5214 | 1033.5223 | 0.87 | 739(100),901(88),871(17) | Ophinpogoside A/isomer |
| 54 | 22.83 | C_50_H_81_O_22_ | 1033.5214 | 1033.5216 | 0.19 | 739(100),901(82),871(15) | Ophinpogoside A/isomer |
| 55 | 23.14 | C_45_H_73_O_18_ | 901.4791 | 901.4791 | 0.00 | 739(100) | Protobioside/isomer |
| 56 | 23.38 | C_45_H_73_O_18_ | 901.4791 | 901.4807 | 1.77 | 739(100) | Protobioside/isomer |
| 57^Δ^ | 23.92 | C_37_H_63_O_11_ | 683.43649 | 683.4376 | 1.62 | 637(100),475(63） | Ginsenoside Rh_1_ |
| 58^Δ^ | 24.02 | C_54_H_91_O_23_ | 1107.5946 | 1107.5944 | -0.18 | 783(100),621(28),766(12),460(12) | Ginsenoside Rb_1_ |
| 59 | 24.40 | C_37_H_63_O_11_ | 683.4365 | 683.4375 | 1.46 | 637(100),475(63) | 20(R)-ginsenoside Rh_1_ |
| 60* | 24.56 | C_54_H_91_O_24_ | 1123.5895 | 1123.5906 | 0.98 | 1077(100),783(12) | M-ginsenoside Rb_2_ |
| 61^Δ^ | 24.56 | C_53_H_89_O_22_ | 1077.5860 | 1077.5836 | -2.23 | 945(100),783(55),621(23),459(20) | Ginsenoside R_C_ |
| 62 | 25.02 | C_45_H_73_O_17_ | 955.4889 | 955.4908 | 1.99 | 793(100),613(34),523(20),569(12) | Ginsenoside R_0_ |
| 63 | 25.22 | C_53_H_89_O_22_ | 1077.5860 | 1077.5830 | -2.78 | 945(100),783(61),765(14),621(11) | Ginsenoside Rb_3_ |
| 64* | 25.23 | C_54_H_91_O_24_ | 1123.5895 | 1123.5906 | 0.98 | 1077(100),783(33),561(27) | Ginsenoside Rb_2_ |
| 65 | 25.72 | C_37_H_63_O_11_ | 683.4365 | 683.4365 | 0.00 | 637(100),475(63) | Ginsenoside F_1_ |
| 66^Δ*^ | 26.47 | C_49_H_83_O_20_ | 991.5472 | 991.5480 | 0.81 | 945(100) | Ginsenoside Rd |
| 67 | 26.56 | C_42_H_65_O_14_ | 793.4377 | 793.4382 | 0.63 | 631(100) | Zingibroside R_1_ |
| 68^Δ*^ | 26.63 | C_53_H_85_O_23_ | 1089.5476 | 1089.5477 | 0.09 | 1044(100),1070(25),1072(12) | Jujube acid saponin B |
| 69 | 27.74 | C_15_H_9_O_4_ | 253.0495 | 253.0507 | 4.74 | 209(100),180(31),210(29),253(17),181(16) | Chrysin |
| 70 | 29.87 | C_44_H_69_O_17_ | 869.4529 | 869.4546 | 1.96 | 737(100),819(4) | Pennogenin3-O-α-L-rhamnopyranosyl-(1→2)-[α-L-rhamnopyranosyl-(1→4)]-β-D-glucopyranoside |
| 71 | 30.18 | C_42_H_69_O_12_ | 765.4766 | 765.4800 | 4.44 | 619(100) | Ginsenoside F_4_ |
| 72 | 30.70 | C_42_H_69_O_12_ | 765.4766 | 765.4796 | 3.92 | 619(100) | Ginsenoside Rg_6_ |
| 73 | 30.73 | C_37_H_61_O_10_ | 665.4252 | 665.4269 | 2.55 | 619(100) | Ginsenoside RK_3_/isomer |
| 74* | 30.75 | C_43_H_73_O_15_ | 829.4944 | 829.4940 | -0.48 | 783(100),622(93),459(18) | Ginsenoside F_2_ |
| 75 | 31.36 | C_37_H_61_O_10_ | 665.4252 | 665.4274 | 3.31 | 619(100) | Ginsenoside Rh_4_/isomer |
| 76 | 32.82 | C_43_H_73_O_15_ | 829.4944 | 829.4956 | 1.45 | 783(100) | Ginsenoside Rg_3_ isomer |
| 77 | 33.00 | C_42_H_71_O_13_ | 783.4896 | 783.4891 | -0.64 | 621(100),459(72) | 20(S) -Ginsenoside Rg_3_ |
| 78 | 33.41 | C_42_H_71_O_13_ | 783.4896 | 783.4895 | -0.13 | 621(100),459(66) | 20(R) -Ginsenoside Rg_3_ |
| 79^Δ^ | 33.44 | C_43_H_73_O_15_ | 829.4944 | 829.4940 | -0.48 | 783(100),622(93) | Ginsenoside Rg_3_ |
| 80 | 35.43 | C_19_H_15_O_6_ | 339.0863 | 339.0874 | 3.24 | 311(100),218(24),205(14),217(13),179(13) | Methyl Ophiopogon High Isoflavone A |
| 81 | 36.39 | C_19_H_17_O_6_ | 341.1020 | 341.1029 | 2.64 | 206(100),178(64),207(43),205(22),179(12) | Methyl Ophiopogon flavanone A |
| 82 | 37.16 | C_19_H_19_O_5_ | 327.1227 | 327.1238 | 3.36 | 206(100),178(26),207(5) | Methyl Ophiopogon flavanone B |
| 83 | 38.31 | C_42_H_69_O_12_ | 765.4756 | 765.4789 | 4.31 | 603(100) | Ginsenoside Rk_1_ |
| 84 | 38.87 | C_42_H_69_O_12_ | 765.4756 | 765.4783 | 3.53 | 603(100) | Ginsenoside R_S5_ |

^Δ^ identified using the reference substance; * [M+HCOO]^-^ ion
